# Synthesis and Antitumor Activity of Doxycycline Polymeric Nanoparticles: Effect on Tumor Apoptosis in Solid Ehrlich Carcinoma

**DOI:** 10.3390/molecules25143230

**Published:** 2020-07-15

**Authors:** Ahmed R. Gardouh, Mohammed A. Attia, Eman T. Enan, Alaaeldeen M. Elbahaie, Rania A. Fouad, Mohamed El-Shafey, Amal M. Youssef, Suliman Y. Alomar, Zinab Abd-Elhady Ali, Sawsan A. Zaitone, Mona K.E. Qushawy

**Affiliations:** 1 Department of Pharmaceutics and Industrial Pharmacy, Faculty of Pharmacy, Suez Canal University, Ismailia 41522, Egypt; Ahmed_mahmoud@pharm.suez.edu.eg; 2 Department of Pharmacy, Faculty of Pharmacy, Jadara University, 21110 Irbid, Jordan; 3 Department of Clinical Pharmacology, Faculty of Medicine, Mansoura University, Mansoura 35516, Egypt; dr_moh_sas@mans.edu.eg; 4 Department of Pharmacology, College of Medicine, Almaarefa University, 71666 Riyadh 11597, Saudi Arabia; 5 Department of Pathology, Faculty of Medicine, Mansoura University, Mansoura 35516, Egypt; emanenan@mans.edu.eg; 6 Department of Clinical Oncology and Nuclear Medicine, Faculty of Medicine, Suez Canal University, Ismailia 41522, Egypt; abahaie@msn.com; 7 Medical Biochemistry Department, Faculty of Medicine, Zagazig University, Zagazig 44519, Egypt; rfouad@mcst.edu.sa; 8 Department of Clinical Biochemistry and Genetics, College of Medicine, Almaarefa University, 71666 Riyadh 11597, Saudi Arabia; 9 Physiological Sciences Department, Fakeeh College for Medical Sciences, 2537 Jeddah, Saudi Arabia; dr_shaf3y@mans.edu.eg; 10 Department of Anatomy and Embryology, Faculty of Medicine, Mansoura University, Mansoura 35516, Egypt; 11 Department of Physiology, Faculty of Medicine, Suez Canal University, Ismailia 41522, Egypt; amalyoussef@med.suez.edu.eg; 12 Doping Research Chair, Department of Zoology, College of Science, King Saud University, Riyadh 11495, Saudi Arabia; 13 Clinical Skills Lab Department, College of Medicine, Imam Abdulrahman Bin Faisal University, P.O. 1982, Dammam 31441, Saudi Arabia; zaabddelhady@iau.edu.sa; 14 Department of Pharmacology and Toxicology, Faculty of Pharmacy, Suez Canal University, Ismailia 41522, Egypt; 15 Department of Pharmacology and Toxicology, Faculty of Pharmacy, University of Tabuk, Tabuk 71491, Saudi Arabia; 16 Department of Pharmaceutics, Faculty of Pharmacy, University of Tabuk, Tabuk 71491, Saudi Arabia; 17 Department of Pharmaceutics, Faculty of Pharmacy, Sinai University, El-Arish, North Sinai 45511, Egypt

**Keywords:** apoptosis, doxycycline, solid Ehrlich carcinoma, female mice, hydroxypropyl methyl cellulose (HPMC), polymeric nanoparticles

## Abstract

Objectives: The aim of this study was to prepare doxycycline polymeric nanoparticles (DOXY-PNPs) with hope to enhance its chemotherapeutic potential against solid Ehrlich carcinoma (SEC). Methods: Three DOXY-PNPs were formulated by nanoprecipitation method using hydroxypropyl methyl cellulose (HPMC) as a polymer. The prepared DOXY-PNPs were evaluated for the encapsulation efficiency (EE%), the drug loading capacity, particle size, zeta potential (ZP) and the in-vitro release for selection of the best formulation. PNP number 3 was selected for further biological testing based on the best pharmaceutical characters. PNP3 (5 and 10 mg/kg) was evaluated for the antitumor potential against SEC grown in female mice by measuring the tumor mass as well as the expression and immunohistochemical staining for the apoptosis markers; caspase 3 and BAX. Results: The biological study documented the greatest reduction in tumor mass in mice treated with PNP3. Importantly, treatment with 5 mg/kg of DOXY-PNPs produced a similar chemotherapeutic effect to that produced by 10 mg/kg of free DOXY. Further, a significant elevation in mRNA expression and immunostaining for caspase 3 and BAX was detected in mice group treated with DOXY-PNPs. Conclusions: The DOXY-PNPs showed greater antitumor potential against SEC grown in mice and greater values for Spearman’s correlation coefficients were detected when correlation with tumor mass or apoptosis markers was examined; this is in comparison to free DOXY. Hence, DOXY-PNPs should be tested in other tumor types to further determine the utility of the current technique in preparing chemotherapeutic agents and enhancing their properties.

## 1. Introduction

Cancer is one of the most important causes of death worldwide [1]. It is characterized by uncontrolled cell proliferation and growth. Ehrlich carcinoma is a mouse breast cancer cell line which is highly similar to the human tumors and acquires high sensitivity to diverse types of antitumor agents [2]. Ehrlich carcinoma is characterized by high tumor growth rate, possibility of transplantation [3] and wide utility for detecting antitumor activity of newly formulated molecules [4].

Doxycycline (DOXY) was first approved by the FDA as an antibacterial agent in 1960s [5]. DOXY has 100% oral bioavailability with long (18–22 h) half-life [6]. The mechanism of action of DOXY involves inhibition of the bacterial protein synthesis through binding to the active aminoacyl-tRNAs with the A-site at 30S subunit of bacterial ribosomes [7]. In mammalian cells, the 28S subunit of mitochondrial ribosomes resembles the 30S subunit of bacterial ribosomes. Thus, the side effects of DOXY involves inhibition of mitochondrial biogenesis due to the similarity between the mitochondrial ribosomes and bacterial ribosomes [8]. This side effect has been repurposed as a therapeutic option in the field of oncology aiming to inhibit the mitochondrial biogenesis in cancer cells [9]. During the clinical use, the adverse effects of DOXY are rare including thrombocytopenia, anemia and neutropenia [10].

Nanotechnology is commonly applied nowadays in many disease therapies [11]. The polymeric nanoparticles (PNPs) are commonly used to improve the therapeutic effect of a wide variety of hydrophilic and hydrophobic dugs by enhancing their solubility, bioavailability and retention time [12]. The PNPs have advantages over the conventional drugs; they provide decreased drug toxicity, enhanced targeting to the specified tissue [13], increased drug efficacy, protection for the drug from degradation by biological fluids, controlled drug release [14] and enhanced drug absorption and bioavailability [15]. PNPs can be prepared from different drugs for treating several diseases like diabetes [16], fungal infections [17], cardiovascular diseases [18] and cancers [19].

Some previous studies highlighted the importance of preparing DOXY in nanoformulations. For example, DOXY-PNPs were prepared to enhance its antibacterial activity against some types of bacteria [20] or to reduce its toxicity [21]. Meanwhile, as an antitumor agent, some types of DOXY nanoparticles were prepared to enhance its penetration into cancer cells [22], augment its anticancer activity [23] or improve drug delivery and efficacy [24,25] however, DOXY-PNPs were not designed before.

The aim of this study was to formulate, for the first time, DOXY-PNPs by the nanoprecipitation method using the hydroxypropyl methyl cellulose (HPMC) polymer. This study monitored the possible boosting for the antitumor potential of DOXY-PNPs in comparison to the free drug in an in-vivo model of inoculated solid tumors focusing on testing the ability of the therapeutic interventions to induce tumor apoptosis.

## 2. Results 

Three formulations of DOXY-PNPs were prepared by nanoprecipitation technique using HPMC as a polymer. The composition of the prepared DOXY-PNPs is presented in Table 1. The prepared formulations were then evaluated for encapsulation efficacy % (EE%), loading capacity % and particle size. In addition, poly dispersity index (PDI), zeta potential (ZP), transmission electron microscopy (TEM) and in vitro release of DOXY were investigated and helped to select the best formulation. 

### 2.1. Estimation of Encapsulation Efficiency and Loading Capacity of DOXY-PNPs

As shown in Table 2, the range of EE% of the prepared DOXY-PNPs was from 42.15 ± 0.84% to 84.65 ± 0.93%. The range of the drug loading capacity % was from 30.29 ± 0.15% to 66.13 ± 0.36 %. The EE% and drug loading capacities (%) were significantly different among the three DOXY-PNP preparations; PNP3 showed the greatest values (*p* < 0.05, Table 2). Hence, the EE% and drug loading capacity % were affected by drug polymer ratio. 

### 2.2. The Particle Size, ZP and PDI of DOXY-PNPs

**Figure 1:**
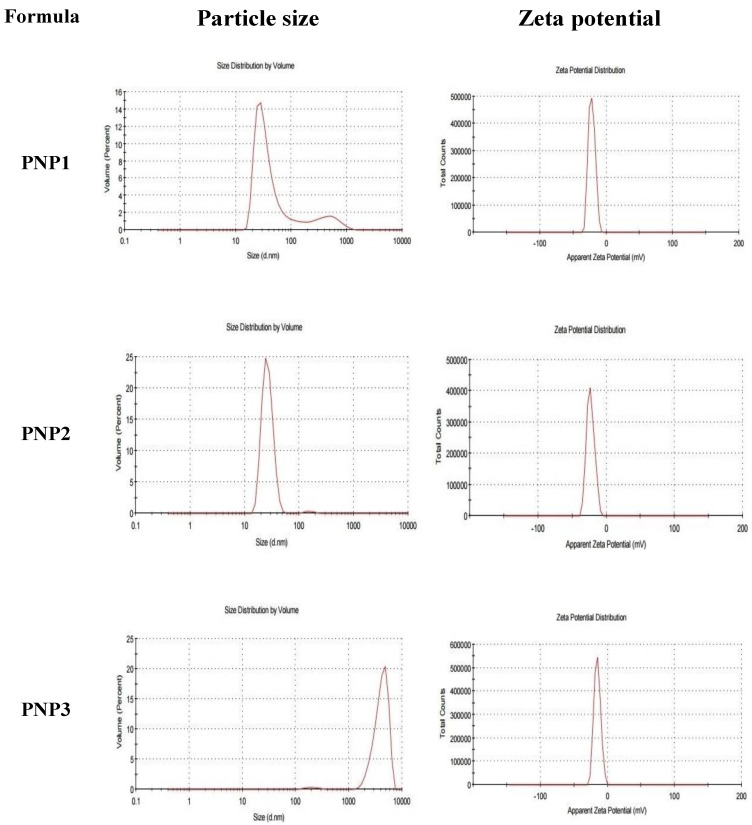
The particle size and zeta potential of the prepared DOXY-PNPs.

The particle size of each formulation was determined by zeta seizer in term of Z-average diameter. As shown by Figure 1 and Table 2, the particle size of the prepared DOXY-PNPs was in the submicronized range as the measured particle size range was minimally 203.6 ± 1.4 nm and 615.3 ± 8.3 nm maximally. For PNP1 the results of peaks were peak 1 at 40.9 nm with percent 85.7% and peak 2 at 474.5 nm with percent 14.3 % and Z-average was 203.6 ± 1.4 nm as obtained from zeta seizer. Similar results in cases of PNP2 where peak 1 at 155.9 nm with percent 1.1 % and peak 2 at 26.59 nm with percent 98.9 % and Z-average was 489.7 ± 6.7 nm, and for PNP3 the Z-average was 615.3 ± 8.3 nm which is the average of peak 1 at 4145 nm with percent 98.4 % and peak 2 at 205.9 nm at 1.6%.

It was found that the particle size was increased by increasing the drug polymer ratio, where PNP1 had the smallest particle size and PNP3 had the highest value of particle size. 

Zeta potential (ZP) is the electric charge that developed on the interface between solid particles and the liquid medium. The high value of ZP of the dispersed particles gives indication about high stability of the dispersion system. The values of ZP ranged from −15.1 ± 4.84 to −23 ± 5.68 mv as appeared in Figure 1 and Table 2, without significant differences between the three PNP preparations. All the prepared DOXY-PNPs exhibited negative ZPs, which indicates high stability for the prepared DOXY-PNPs due to low propensity for aggregation. 

PDI is a value which gives indication about the heterogeneity of the particle size of the prepared NPs. The PDI values were 0.43 ± 0.02, 0.85 ± 0.05, and 1.00 ± 0.04 for PNP1, PNP2 and PNP3, respectively, with significant differences between them (*p* < 0.05, Table 2). 

### 2.3. The In-Vitro Release of DOXY from the Prepared PNPs

**Figure 2:**
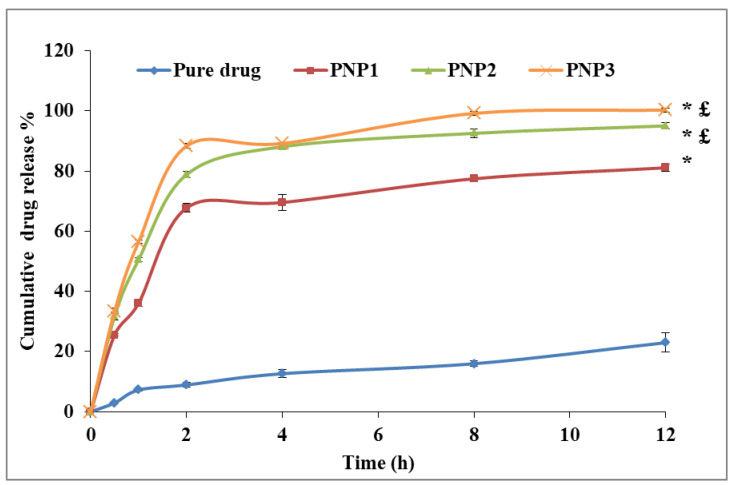
The cumulative in vitro release of DOXY from the different formulas. Data for the cumulative release were measured over 12 h and presented as mean ± SD. Statistical analysis for the last data point was done using one-way ANOVA followed by Bonferroni’s post-hoc test at probability value < 0.05, * Compared to the free drug, **^£^** Compared to PNP1.

The in-vitro release study of the prepared DOXY-PNPs was done using the dialysis bag method. The percentage of the released DOXY was plotted against time as shown by Figure 2. It was observed that the release of DOXY from the prepared PNPs was much higher than the release from the pure drug. Similar to the above-mentioned results, the drug release was affected by drug polymer ratio, where PNP3 > PNP2 > PNP1.

### 2.4. The Surface Morphology of the Prepared DOXY-PNPs

**Figure 3:**
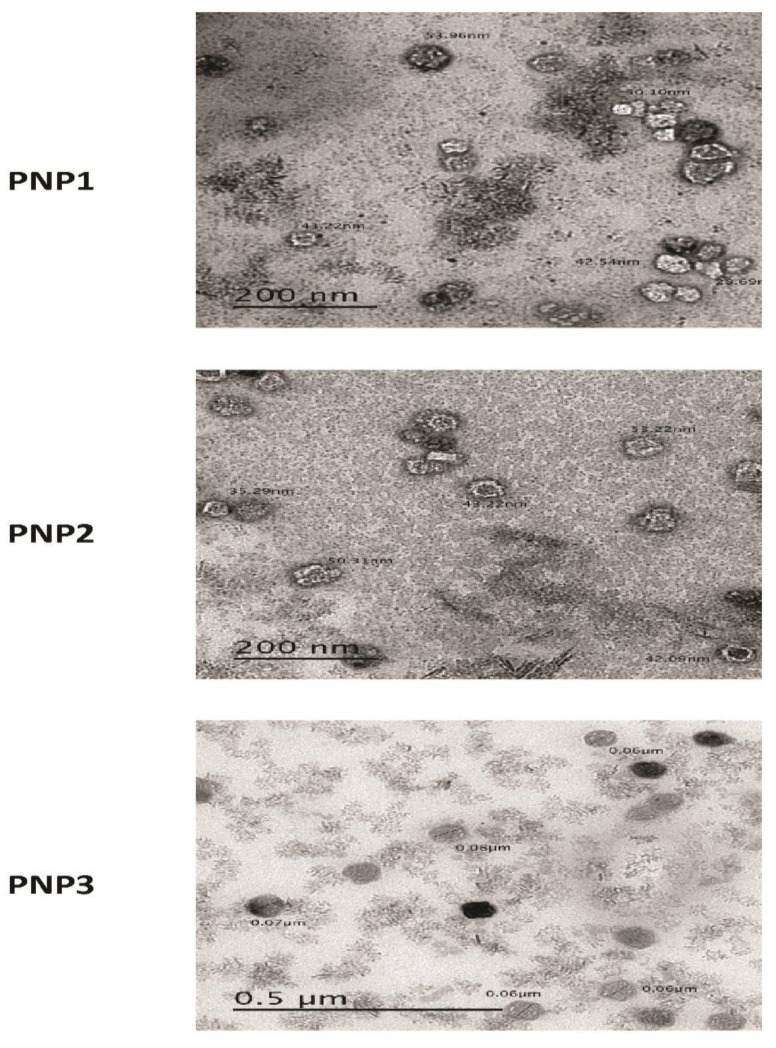
Transmission electron microscope photographs of prepared DOXY–PNPs.

The morphology of the prepared DOXY-PNPS was examined by TEM. The images of the prepared DOXY-PNPs showed their spherical shape with a smooth surface (Figure 3). As shown by the TEM images, the particle size of the 3 prepared PNPs are smaller than the values of the particle size measured by Zetasizer.

### 2.5. Selecting the Best Formulation

From the previous results, formula PNP3 showed the highest EE%, drug loading capacity and the best drug release therefore, it was selected to be tested for the biological activity in the in-vivo study.

### 2.6. In-Vivo Pharmacological Activity for DOXY-PNP3

#### 2.6.1. Survival Data

Each of the experimental groups started with 6 mice at the beginning of the study but no mortality was observed during the examination period. Using the survival criterion depending on the tumor mass, none of the mice showed tumor masses greater than 1 g (the maximum mass was 952 mg), therefore none of them were considered “dead” and the survival % was registered as 100% in all the experimental groups. 

#### 2.6.2. Mass of the Solid Tumors

**Figure 4:**
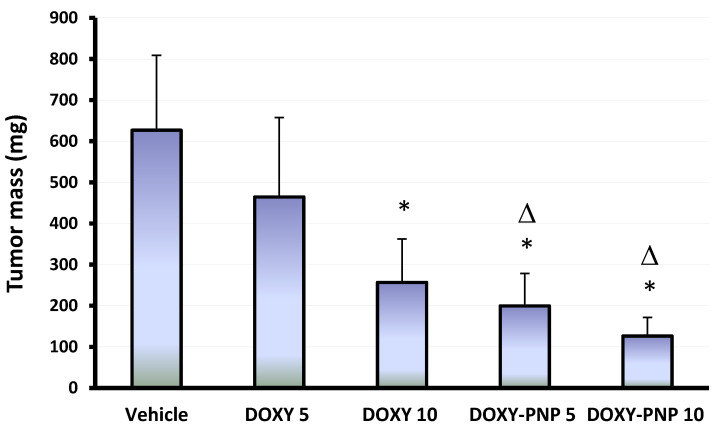
The mass of the solid tumors in the experimental groups. Column chart demonstrates the mean value of tumors for each mouse ± SD. Data were analyzed using multivariate ANOVA followed by Bonferroni’s post-hoc test at probability value < 0.05. * Versus vehicle and ^Δ^ versus DOXY 5 mg/kg, *n* = 6.

Multivariate ANOVA indicated significant differences between the study groups. The current experiment showed that solid Ehrlich carcinomas (SECs) grown in the vehicle treated group had the largest mass among the study groups (626.8 ± 181.8 mg, Figure 4). Post-hoc analysis indicated that treatment with 5 mg/kg of free DOXY did not reduce the mass of the solid tumors significantly. However, the large dose of free DOXY led to a significant decline in the tumor mass compared with the vehicle group but did not reduce the tumor mass significantly compared to the 5 mg/kg DOXY group (Figure 4). Both doses of DOXY-PNPs reduced the mass of the solid tumors compared with the vehicle treated group. The antitumor effect of 10 mg/kg of DOXY-PNPs was greater than that produced by 5 and 10 mg/kg of free DOXY (Figure 4). 

#### 2.6.3. Quantitative Polymerase Chain Reaction for Tumoral mRNA Expression of BAX and Caspase 3

**Figure 5:**
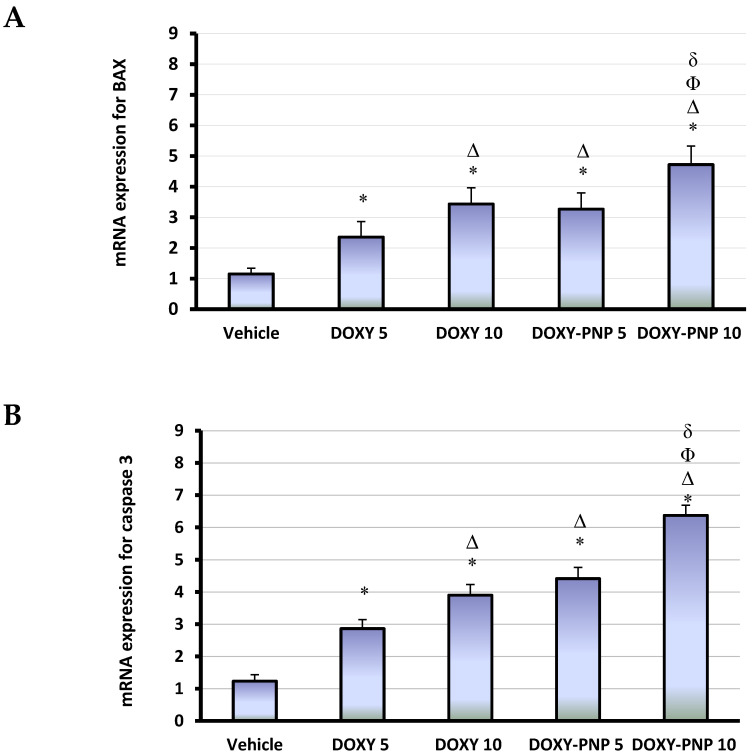
The tumoral mRNA expression for BAX and caspase 3. Column chart demonstrates the mean value of BAX (**A**) and caspase 3 (**B**) expression for each mouse ± SD. Data were analyzed using multivariate ANOVA followed by Bonferroni’s post-hoc test at probability value < 0.05. * Versus vehicle and ^Δ^ versus DOXY 5 mg/kg, ^Φ^ versus DOXY 10 mg/kg, ^δ^ versus DOXY-PNP 5 mg/kg, *n* = 6.

Apoptotic events comprise fragmentation of DNA, condensation of nuclei and cell shrinkage [26]. Activating caspase leads to cleavage to particular proteins and is crucial in the process of apoptosis [27]. BAX is a pro-oncoprotein that promotes tumor cell death when upregulated in the tumor cells [28]. Tumor samples from the 5 and 10 mg/kg DOXY treated groups showed significant increases in BAX (2.05-fold and 2.98-fold) and caspase 3 (2.33-fold and 3.17-fold) expressions. Similarly, 5 and 10 mg/kg of DOXY-PNPs showed increased BAX (2.84-fold and 4.10-fold, Figure 5A) and caspase 3 (3.59-fold and 5.17-fold, Figure 5B). 

The effect of treatment with 5 mg/kg of DOXY-PNPs was significantly higher than that produced by 5 mg/kg of free DOXY (Figure 5A). Similarly, the effect of 10 mg/kg of DOXY-PNPs was significantly greater than that detected by 10 mg/kg of free DOXY (Figure 5B). 

#### 2.6.4. Assessment of Histologic Features and Tumor Necrosis in Sections Stained with H&E

**Figure 6:**
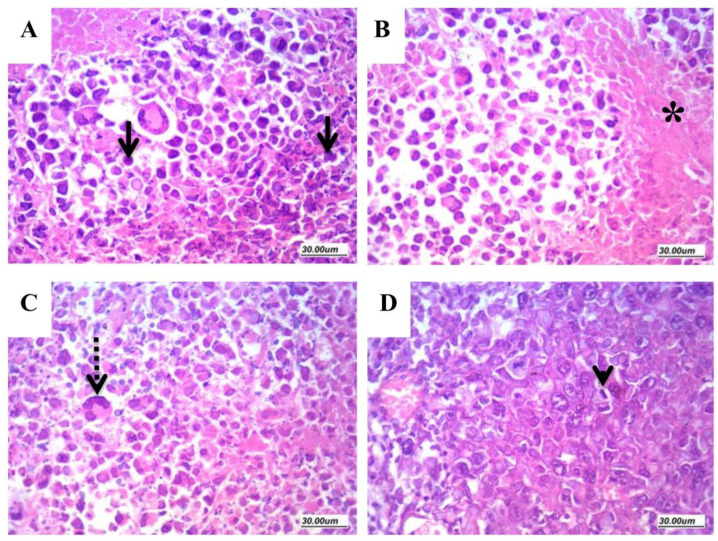
Histopathologic features of solid Ehrlich carcinomas. Sections from the solid tumors stained with H&E (×400) showing: (**A**) apoptosis (arrows); (**B**) necrosis (star); (**C**) giant cells (dashed arrow); and (**D**) mitosis (arrow head).

The sections prepared from solid tumors of the vehicle group (H&E staining) showed high grade malignant growth formed of markedly atypical cells arranged in sheets with minimal foci of necrosis. Frequent atypical mitoses and scattered bizarre and giant cells were detected (Figure 6).

**Figure 7:**
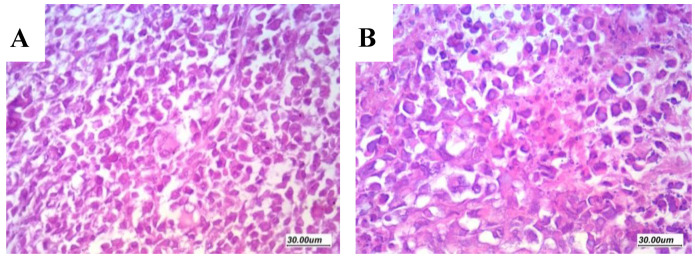

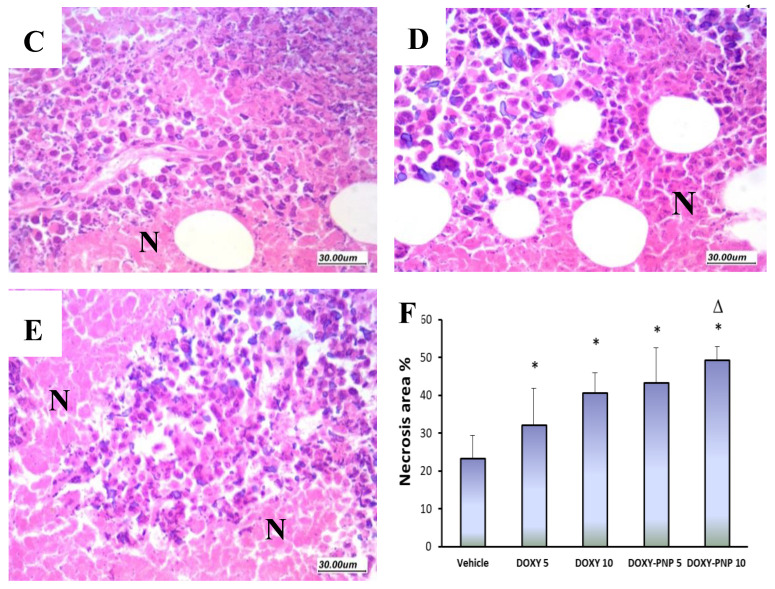
Extent of tumor necrosis in solid Ehrlich tumors. Sections from the solid tumors stained with H&E (×100) showing (**A**) the vehicle group with minimal necrosis within viable tumor tissue, compared to wide areas of tumor necrosis in the groups treated by either free DOXY (5 and 10 mg/kg) (**B**,**C**) or DOXY-PNP (5 and 10 mg/kg) (**D**,**E**); N—refers to necrosis area. (**F**) Column chart demonstrating the mean necrotic area ± SD. Data were analyzed using multivariate ANOVA followed by Bonferroni’s post-hoc test at probability value < 0.05. * Versus vehicle, ^Δ^ versus DOXY 5 mg/kg, ^Φ^ versus DOXY 10 mg/kg, ^δ^ versus DOXY-PNP 5 mg/kg, *n* = 6.

Examination of sections from the DOXY-treated mice revealed wider areas of necrosis if compared to the vehicle-treated mice. Even though the free DOXY treated tumors showed evident tumor necrosis and apoptosis, tumors of the DOXY-PNPs -treated groups remarkably exhibited substantial areas of necrosis and prominent apoptosis (Figure 7A). The 10 mg/kg DOXY-PNP group showed greater necrosis area than the 5 mg/kg free DOXY group (Figure 7B).

#### 2.6.5. BAX and Caspase 3 Immunostaining in the Solid Tumors

**Figure 8:**
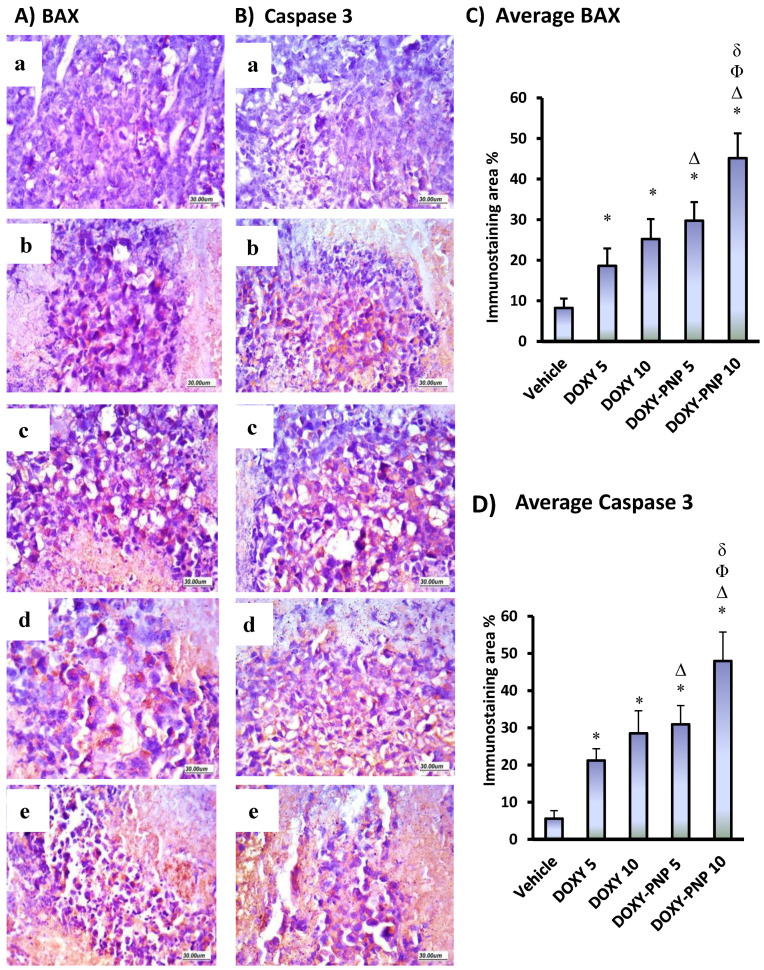
Immunohistochemical staining for BAX and caspase 3 in solid tumors. (**A**) Images for tumor specimens stained for BAX protein in different groups (**a**—vehicle; **b**—DOXY 5; **c**—DOXY; **d**—DOXY-PNPs 5; **e**—DOXY-PNPs 10), (**B**) images for tumor specimens stained for caspase 3 in different groups (**a–e**), (**C**,**D**) column charts demonstrating the mean area of staining ± SD for BAX and caspase 3. Data were analyzed using multivariate ANOVA followed by Bonferroni’s post-hoc test at probability value < 0.05. * Versus vehicle, ^Δ^ versus DOXY 5 mg/kg, ^Φ^ versus DOXY 10 mg/kg, ^δ^ versus DOXY-PNP 5 mg/kg, *n* = 6.

As demonstrated in Figure 8, expression of both BAX and caspase 3 in immunohistochemically stained sections was mostly of cytoplasmic localization. Regarding BAX expression, it was significantly higher in tumors from animals that received 10 mg/kg of free DOXY and both doses of DOXY-PNP as compared to the vehicle group. The high dose of free DOXY enhanced BAX staining in comparison to the vehicle group and 5 mg/kg free DOXY group. However, a dose dependent increase in staining for BAX was detected in the mice groups received 5 and 10 mg/kg of DOXY-PNPs. Likewise, caspase 3 staining was greater in experimental groups treated with free DOXY and DOXY-PNPs than in the vehicle group. Evidently, treatment with the high dose DOXY-PNPs resulted in significantly greater caspase 3 expression compared to all the experimental groups (Figure 8).

#### 2.6.6. Simple Linear Regression Analysis for the Tumor Data

**Figure 9:**
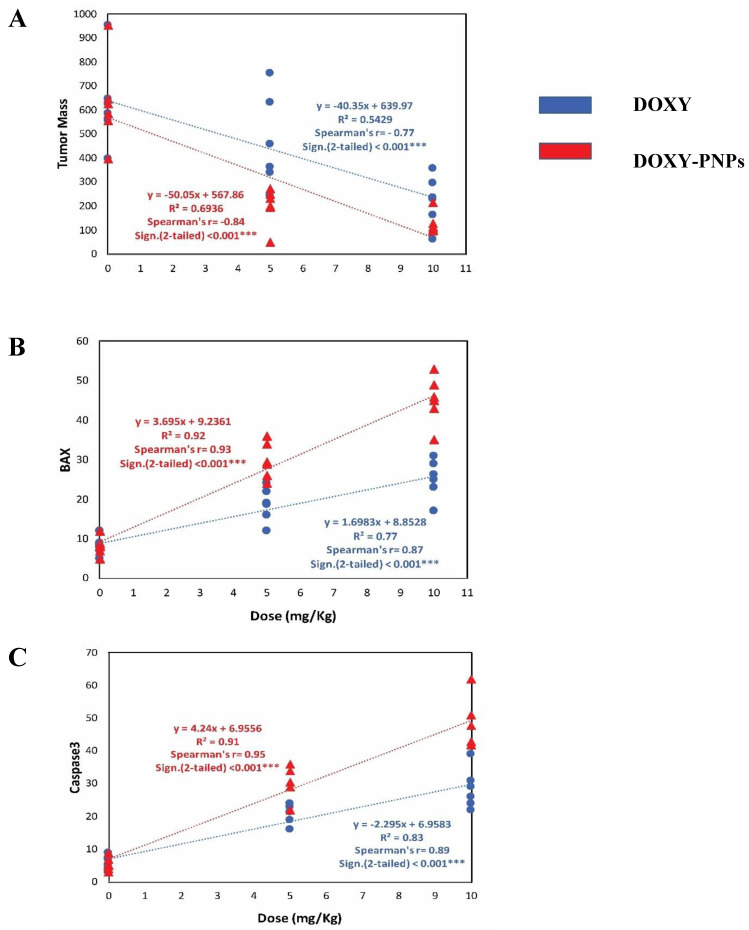
Regression trend-line for correlation between the drug preparations and tumor markers. The relationship between dose (mg/kg) on x axis and tumor mass (**A**), BAX immunostaining area (**B**) and caspase immunostaining area (**C**) on Y axis. *r* = Spearman’s correlation coefficient, *** *P* < 0.001, *n* = 6.

The current data were evaluated by simple linear regression of tumor mass in relation to the dose of DOXY and DOXY-PNPs (Figure 9). Linear regression analysis and trend-line indicated that free DOXY and DOXY-PNPs strongly and significantly decreased tumor masses (*r* = −0.77, and *r* = −0.84, respectively). However, DOXY-PNPs strongly decreased tumor masses than free DOXY (Figure 9A). BAX was strongly elevated with increased doses of free DOXY (*r* = 0.87) and DOXY-PNPs (*r* = 0.93) as revealed by Spearman’s correlation and simple linear regression (Figure 9B). Immunohistochemistry of caspase 3 was greater with increasing doses of DOXY (*r* = 0.89) and DOXY-PNPs (*r* = 0.95) as revealed by Spearman’s correlation and simple linear regression (Figure 9C).

## 3. Discussion

The current study aimed to test the antitumor potential of newly synthetized DOXY-PNPs against SECs grown in female mice. Nanoformulations of DOXY reduced the particle size, enhanced the drug release and hence, superior antitumor potential was expected. The antitumor effect was measured in terms of tumor mass, necrosis and apoptosis.

### 3.1. Characterization of DOXY-PNPs

In the current study the DOXY-PNPs were prepared by nanoprecipitation technique and evaluated for EE% and the loading capacity. We found that both EE% and drug loading capacity % increased by raising the drug polymer ratio PNP3 > PNP2 > PNP1. In agreement, both EE% and the loading capacity of the formulations were reported previously to change according the drug polymer ratio [29]; the increase in these 2 pharmaceutical indicators is correlated by the rise in the drug polymer ratio; this may be related to high drug miscibility in the polymer and the drug polymer interaction [30]. High drug miscibility in the polymer leads to higher EE% and drug loading capacity. These results agree with those reported previously [31]; authors prepared carvedilol NPs and found that EE% was improved with higher drug concentrations. Another research group synthetized glibenclamide PNPs using the solvent displacement method and found that the drug loading and EE% were in a direct correlation to the drug polymer ratio [32]. The drug loading of DOXY-PNPs in the current study was increased with higher drug polymer ratio which indicates greater amount of the loaded drug. This observation is in agreement with that documented previously [33].

The increase in particle size of the prepared DOXY-PNPs was dependent on increasing the drug to polymer ratio from 1:2 to 2:1. This may be attributed to the higher amount of the encapsulated drug in the polymer core of the PNPs by increasing the drug to polymer ratio; this was evidenced by the EE% results [33]. These results agree well with those reported a similar this relation for poly-ε-caprolactone NPs prepared using the solvent displacement method [29]. Consistently, the particle size of glibenclamide NPs was reported to increase by increment in the drug to polymer ratio [32].

The ZP of the prepared DOXY-PNPs was found low (range from −15.1 ± 4.84 to −23 ± 5.6 mV). The low value of ZP may be attributed to the adsorption of non-ionic surfactant (Tween 80) onto the surface of prepared NPs resulting in partial screening of the surface charge and slight reduction in the value of ZP. This result is in accordance with the results obtained previously [29]; authors of this article synthetized poly-ε-caprolactone NPs by solvent displacement method by two non-ionic surfactants (Pluronic^®®^ F-68 and Tween 60) and found that the measured ZP values were significantly augmented after centrifugation of nanosuspension and redispersion in water where the ZP increased from −21.2 ± 0.64 mV to −30.5 ± 0.7 and from −11.7 ± 0.32 to −25.1 ± 0.40 mV in the case of Pluronic^®®^ F-68 and Tween 60, respectively. The authors concluded that the surface charge was screened by the nonionic surfactant. The results of PDI values of the prepared DOXY-PNPs were smaller than 1; which is indicative for the homogeneity of the particle size [34,35]. López-López et al., who prepared levofloxacin loaded polymeric nanoparticles found that PDI value ranged from 0.42 ± 0.05 to 0.91 ± 0.09 [36]. This result is in agreement with that obtained by Suksiriworapong et al., who prepared ibuprofen and indomethacin PNPs by nanoprecipitation method and documented that the particle size and PDI increased by increasing the drug to polymer ratio [33]. 

According to the results of the in vitro release study, it was observed that the release of DOXY from the prepared PNPs was much higher than the free pure drug; this may be explained by the increased saturation solubility of DOXY when prepared as PNPs [32]. It was found that the cumulative amount of released DOXY increased by elevating the drug to polymer ratio; this means that PNP3 with drug polymer ratio (2:1) presented the highest rate of release as shown in Figure 2. These outcomes may be attributed to the lower proportion of the polymer with increasing the drug polymer ratio resulted in augmented drug diffusion to the dissolution medium [37]. These results are in full concordance with those obtained by Behera et al. [38], who prepared glibenclamide NPs and found that the release rate increases by increasing the drug polymer ratio.

In addition, the PNPs looked smaller when observed with the TEM, in comparison to the measured particle size which may be attributed to PNPs dehydration through preparation of TEM samples [37].

### 3.2. In-Vivo Pharmacological Activity of DOXY-PNPs

Doxycycline is a second-generation tetracycline derivative with wide antimicrobial spectrum. Its antibiotic properties are attributed to its ability to inhibit the microbial protein synthesis by interfering with amino acyl tRNA-30S ribosome binding [39]. Many reports have demonstrated that DOXY has antitumor properties by inhibiting cell growth and inducing apoptosis in several kinds of malignancies both in-vitro and in animal models [40,41]. These cytotoxic effects of DOXY and some other tetracycline derivatives are often obtained at high doses, which may be the reason for the adverse reactions detected in some clinical trials testing their anticancer effect [40,41]. A promising strategy to overcome this problem is to use PNPs, which act to enhance the concentration of anticancer drugs inside tumor cells and diminish their systemic toxic effect [42,43].

In several previous studies, the antibacterial activity of DOXY has been reported to be enhanced by encapsulation in PNPs [44]. However, similar effect on DOXY’s antitumor activity has not been tested before. Therefore, in the current study, we assessed the effectiveness of the formulated DOXY-PNPs in suppressing tumor growth and inducing apoptosis/necrosis in solid Ehrlich carcinomas. By assessing solid tumor masses of the study groups, we demonstrated that in comparison to free DOXY, DOXY-PNPs produced more significant reductions in tumor masses; the antitumor effect of the high dose (10 mg/kg) of DOXY-PNPs was the greatest among all tested formulas. Consistent results in solid Ehrlich carcinoma mouse model have been previously achieved using a nanoform of a chemotherapeutic agent which demonstrated greater efficacy as compared to the free form [2].

The current results indicated greater apoptosis in mice treated with the high dose of DOXY-PNPs; this was matched with the produced suppression in tumor mass. It is known that activating apoptosis process is a key method by which anticancer drugs eliminate malignant cells [45,46]. A family of caspases plays the major role in cell apoptosis after being activated through the extrinsic (FAS receptor) pathway, or intrinsic (mitochondrial) pathway [47]. Particularly, mitochondrial pathway controls apoptosis via cytochrome c release from mitochondria membrane to the cytoplasm. Activating the pro-apoptotic proteins like BAX, is essential for releasing cytochrome c from the mitochondria [48]. In living cells, DOXY transfers to the cytoplasm and accumulates in mitochondria to be metabolized producing stable photo molecules, that disrupt mitochondrial membrane and inhibit mitochondrial ability to synthesize essential proteins [49].

Several mechanisms of DOXY-induced cytotoxicity have been suggested including the induction of apoptosis. In various tumors including pancreatic cancer, colonic cancer and small cell lung carcinoma cells, apoptosis induction by DOXY has been found to involve caspase 3 activation [49,50]. Therefore, we evaluated the effect of free and nano-DOXY on tumor cell apoptosis through assessing the immunohistochemical expression of two important apoptosis-related proteins, BAX and caspase 3. Among our studied groups, animals treated with DOXY-PNPs (5 and 10 mg/kg) showed tumors with strong significant increases in the expression of caspase 3 and BAX in comparison to the vehicle group. The high dose of DOXY-PNPs showed greater tumor apoptosis compared to free DOXY. This indicates that the formulation of DOXY in PNPs improves its pro-apoptotic effect which may be due to the enhanced delivery into tumor cells [51].

In agreement to our results, treatment of Ehrlich carcinoma bearing mice with nano-5-flurouracil in a previous study caused a significant increase in caspase 3 expression when compared with free 5-flurouracil treated mice [2]. Likewise, studies comparing the effect of free-, and nano-5-flurouracil-induced apoptosis in astrocytoma and breast carcinoma cells, have reported the more superior efficacy of the nano-loaded drug [52].

Indeed, various PNP-based chemotherapy studies have reported that loading of chemotherapeutic agents on PNPs resulted in improved entry and sustained localization of drugs in the tumors, which markedly enhanced their anticancer effect [53]. In osteosarcoma, Chen and colleagues formulated ifosfamide-loaded poly (lactic-co-glycolic acid) dextran PNP and used it against “SaOS-2” and “MG63” cancer cells [51]. They reported that these nano-carriers triggered augmented drug delivery and exhibited increased anticancer action and superior apoptotic effect than the free ifosfamide.

Three studies used doxorubicin, another antibiotic chemotherapeutic agent, in doxorubicin-loaded polymer-lipid hybrid NPs (DL-PLHNPs) formulae. One study highlighted the in-vivo efficacy in terms of delaying tumor growth and substantial increase in tumor necrotic areas than the untreated tumors, with minimal systemic toxicity [54]. The second study found enhanced uptake and retention of the drug in two different multidrug resistant breast cancer cell lines compared with free doxorubicin solutions [55]. The third one highlighted that, the polymer–lipid hybrid NP formula of doxorubicin increases cytotoxicity against multidrug-resistant human breast cancer cell lines [56].

In agreement, concurrent delivery of doxorubicin and GG918 (a chemosensitizer) by polymeric lipid hybrid NPs improved therapy of multidrug-resistant breast cancer in acute and long term studies [57]. Another study showed that, multiple layer-by-layer polymeric lipid hybrid NPs amended the FOLFIRINOX (5-fluorouracil, oxaliplatin, irinotecan plus folinic acid) in the treatment of patients with pancreatic cancer and possessed higher antitumor efficacy and minimal adverse effects in comparison to the per se FOLFIRINOX regimen. Furthermore, these NPs were more efficacious than the long-term reference standard, gemcitabine [58].

Some other chemotherapeutic agents prepared in nanoformulations were proven effective compared to their free form. For example, paclitaxel-loaded PEG-PE–based micellar nanopreparations reduced tumor and enhanced apoptosis in-vivo and in-vitro. The animals did not show hepatotoxicity or pathologic changes in the vital organs [59].

In agreement with these studies, synthetized doxorubicin and indocyanine green NPs loaded on poly (lactic-co-glycolic acid)–lecithin–PEG (DINPs) were tested for biological activity in a previous study. The DINPs presented stable spectral characters, excellent stability and good dispersity versus the free drugs. Furthermore, the DINPs showed longer retention time within the tumors [60]. 

## 4. Materials and Methods

### 4.1. Materials

DOXY hyclate in a yellow powder form (lot number 0000042274) was donated by Tabuk Pharmaceutical Company (Tabuk, Saudi Arabia). HPMC (MW = 1250 g) was purchased from Shin-Etsu Company in Tokyo (Japan). Acetone (El-Nasr Chemical Company, Benha, Egypt) and Tween 80 (Kolb distribution Ltd., Hedingen, Switzerland) were utilized in the preparation of the nanoformulations. Other solvents and chemicals were bought in analytical grades.

### 4.2. Synthesis of Doxycycline Polymeric Nanoparticles

#### 4.2.1. Doxycycline Polymeric Nanoparticle Preparation

Three formulations of DOXY loaded PNPs were prepared by nanoprecipitation method [61]. The accurate amount of polymer (HPMC) was dissolved in a water miscible organic solvent (acetone) to form the organic phase with a concentration of 0.8 g % [62]. The aqueous phase was prepared by liquefying the accurate quantity of DOXY in double distilled water containing tween-80 (1% w/v). The addition of the organic phase into the aqueous one was done drop wisely with stirring where the ratio of organic phase to aqueous phase was 1:8. The mixture was undergoing stirring at room temperature using a magnetic stirrer (Brandstead/Thermolyne, Dubuque, IA, USA). The mixture was allowed to stir until the preparation of the NPs where was converted to milk like colloidal dispersion [63]. The residual of organic solvent was removed by exposure to stirring for an overnight at the room temperature [64].

#### 4.2.2. Estimation of DOXY-PNPs Encapsulation Efficiency and Loading Capacity 

The EE% for DOXY in the prepared PNPs was determined by an indirect method [65]. The entrapped drug was separated from the un-entrapped by centrifugation [66] for 30 min at 14,000 rpm (17,979× *g*) using a Hettich centrifuge (Hettich, Mikro 22 R, Germany). The amount of the un-entrapped DOXY in the supernatant was estimated spectrophotometrically at 269 nm utilizing an ultraviolet spectrophotometer (Shimadzu, Japan). The experiment was done in triplicate and the EE% was calculated using the next equation [67]:

(1) $$\mathrm{Encapsulation}\;\mathrm{efficiency}\;\%\;=\frac{\left(\mathrm{Total}\;\mathrm{DOXY}\right)-\left(\mathrm{Un}-\mathrm{entrapped}\;\mathrm{DOXY}\right)}{\mathrm{Total}\;\mathrm{DOXY}}\times 100$$

Additionally, the loading capacity percentage which represents the percentage of the amount of drug (DOXY) in the prepared PNPs was determined. The loading capacity % was measured through the following equation [68]:

(2) $$\mathrm{Loading}\;\mathrm{capacity}\;\%=\frac{\mathrm{Mass}\;\mathrm{ofentrappe}\;\mathrm{DOXY}}{\mathrm{Mass}\;\mathrm{of}\;\mathrm{prepared}\;\mathrm{PNPs}\;}\times 100$$

#### 4.2.3. Determination of Particle Size, Zeta Potential and Polydispersity Index of the Prepared DOXY-PNPs

The particle size, zeta potential (ZP), and polydispersity index (PDI) of all prepared DOXY-PNPs were measured using a zeta seizer (Malvern Instruments Ltd., Malvern, UK) [69]. Each sample of DOXY-PNPs was diluted with distilled water before analysis [70,71].

#### 4.2.4. The Surface Morphology Determination Using Transmission Electron Microscopy

The morphology of the surface of the prepared DOXY-PNPs was determined using TEM (JTEM-2100, JEOL, Tokyo, Japan). Every sample was subjected to dilution with appropriate amount of water then, one drop from each sample was placed on a collodion-coated copper grid [72]. After drying of the samples, they were stained and examined using TEM [73].

#### 4.2.5. The In-vitro Release Study of DOXY from Prepared PNPs

The in-vitro release study of the prepared DOXY-PNPs was done using dialysis bag method [74]. A 4-ml sample from each formulation was transferred to the dialysis bag which then was immersed in 100 mL of simulated gastric fluid (0.1 N HCl, pH = 1.2) for 2 h then substituted with phosphate buffer (pH = 6.8) for the next 10 h. The dissolution medium was kept at 37 ± 1 °C on a magnetic stirrer working at a rate equals 100 rpm. A 1 mL sample was withdrawn at different time intervals to determine the amount of the released drug [75]. The withdrawn samples were filtered after appropriate dilution and analyzed using a UV spectrophotometer at 269 nm [76]. The measurements were done in triplicate and the mean ± SD was calculated. The percentage of the released DOXY was plotted against time in h.

#### 4.2.6. The Selection of the Best Formulation of DOXY-PNPs

The selection of optimized formulation was done based on the highest EE%, drug loading capacity and in-vitro drug release.

### 4.3. The Pharmacological Activity of DOXY-PNPs

#### 4.3.1. Animals

Thirty female Swiss albino mice (body weight = 19–27 g) were procured from Moustafa Rashed Company (Giza, Egypt). Mice were housed in controlled hygienic laboratory settings in an equipped animal facility at the Faculty of Pharmacy, Suez Canal University. Each group of six mice was housed in one cage under normal dark/light cycles and provided with food and water ad libitum. The study protocol and animal care procedures were approved [# 201906RA2] by the institutional research ethics committee at Faculty of Pharmacy, Suez Canal University and maximum efforts were paid off to decrease animal suffering. In addition, the NIH guidelines for the care and use of laboratory animals (8th edition) were followed during the course of the experiment (Albus, 2012). Qualified personnel were responsible for the animal feeding, handling, tumor inoculation and drug delivery throughout the experiment. Any contaminated food or feces were handled regularly [77].

#### 4.3.2. Inoculation of Ehrlich Solid Tumors in Female Mice

The Ehrlich carcinoma cells were supplied by the Cancer Biology Department at the National Cancer Institute in Cairo (Egypt). Under aseptic conditions, the Ehrlich carcinoma cells were prepared and examined for the viability by Trypan blue exclusion method [78] using a light microscope as reported formerly [79]. The cells were prepared in a form of suspension using physiological normal saline with concentration of 25 million cells/1 mL [80]. In the first day, each mouse was inoculated with 0.1 mL of the prepared suspension to induce the solid tumor. The inoculation was done subcutaneously in 2 locations at the ventral side [81].

#### 4.3.3. Experimental Design

After seven days from the inoculation of tumor cells in mice, thirty mice were randomized into five groups of 6 mice. The animals received the following treatment plans.

Group (i): Vehicle group: mice carrying SEC and received the vehicle of DOXY (6 mL/kg by oral tube).

Group (ii and iii): mice carrying SEC received 5 and 10 mg/kg of free DOXY (p.o.), two times per week.

Group (iv and v): mice carrying SEC received 5 and 10 mg/kg of DOXY-PNPs (p.o.), two times per week.

In general, mice received the drugs each Monday and Thursday at 3 p.m. over three weeks.

#### 4.3.4. Sacrification and Separation of Solid Tumors

Three days after completing the previous treatment plans, mice were anesthetized and sacrificed by cervical dislocation. After scarifying the animals, both right and left tumor discs were dissected and weighted. Surviving mice in each group were counted according to a survival criterion. In details, mice with final tumor mass exceeding 1 g were considered as “dead”.

Tumors samples were fixed in paraformaldehyde solution for 16 h and then inserted in paraffin wax. After that, tumor specimens were prepared from the paraffin blocks to be subjected to the histologic staining by hematoxylin and eosin (H&E) and immunohistochemical staining for caspase 3 and the BCl2-associated X-protein (BAX). Tumor specimens were frozen immediately and used in the quantitative polymerase chain reaction PCR assay as described later.

#### 4.3.5. Quantitative polymerase chain reaction (PCR) of caspase 3 and BAX genes

The assay included 2 main steps as follows:

1. RNA Extraction: Homogenized tumor samples from the study groups were lysed and RNAeasy Mini Kit (Qiagen) Kit was used for isolation of total RNA which was examined for both quality and quantity with a dual spectrophotometer (Beckman, Waltham, MA, USA).
2. Real Time PCR: For quantitative determination of mRNAs expression of caspase 3 and BAX, the following procedure was performed. A quantity of 10 ng of the total RNA was directed for the synthesis of cDNA employing the Applied Biosystems high capacity cDNA reverse transcriptase kit (USA). Amplification of the cDNA was then achieved in a 48-well plate by Syber Green I PCR Master Kit (Fermentas) using the Step One instrument (Applied Biosystems, Waltham, MA, USA). Alterations in gene expression were normalized to the mean critical threshold values obtained with GAPDH by the ΔΔCt method [82]. 1 μM of the gene primers was used in the assay and are demonstrated in Table 3.

#### 4.3.6. Histologic Investigation of the Solid Ehrlich Carcinomas

Tumor sections were prepared in 4-µm thick sections and processed for H&E staining for histopathological examination. At least two tumor sections from each animal were compared for tumor cell morphology, mitotic activity, apoptosis and extent of tumor necrosis.

#### 4.3.7. Immunohistochemical Determination of BAX and Caspase 3

For assessment of apoptosis proteins, immunostaining was performed on tumor paraffin blocks. After deparaffinizing sections in xylene and tissue rehydration, heat induced antigen retrieval was carried on. This was followed by incubation overnight with the primary antibodies which are rabbit polyclonal antibody against BAX (1:100, GeneTex, Inc., Irvine, CA, USA) and ready to use rabbit polyclonal antibody against caspase 3 (Thermo Fischer Scientific Anatomical Pathology, Fremont, CA, USA) at 4 °C, according to the instructions of the manufacturer. Then, sections were processed for the conjugation step. Diaminobenzidine was used as a chromogen substrate and hematoxylin was used for nuclear counterstaining.

After examining the slides using a light microscope and photomicrography, the images were analyzed with the ImageJ program (NIH, Bethesda, MD, USA). The proportion of BAX or caspase 3 positive immunostaining was calculated in high-power field images from each animal.

#### 4.3.8. Data Analysis

Data were presented as a mean ± standard deviation. Data related to cumulative release of drug from nanoformulations was checked using one-way ANOVA followed by Bonferroni’s post-hoc analysis. Data of tumor mass and immunohistochemistry for BAX and caspase 3 were analyzed by multivariate ANOVA. Then, one-way ANOVA followed by Bonferroni’s post-hoc test were applied to determine every possible comparison between the study groups. The correlation between the doses of either DOXY or DOXY-PNPs was assessed by Spearman’s correlation and simple linear regression analysis at *p* < 0.05 two tailed significance test. Further, regression trend-line were generated where, doses (x-axis) and tumor mass, caspase 3 or BAX immunostaining on Y-axis. Statistical analyses were carried out using IBM-SPSS version 23.0 for Mac OS [83,84]. The significance of the difference was established at *p*-value < 0.05.

## 5. Conclusion

The current work highlighted the utility of applying polymeric nanotechnology in enhancing the chemopreventive effect of doxycycline against solid tumors in mice. Further studies are needed to ensure the effectiveness, cellular uptake and/or toxicity in other in-vivo and in-vitro models of cancers.

## Figures and Tables

**Table 1:** The composition of prepared doxycycline polymeric nanoparticles (DOXY-PNPs).

Formula Code	Drug: Polymer Mass Ratio	HPMC Concentration	Tween 80 Concentration	Organic: Aqueous Phase
**PNP1**	1:2	0.8 g%	1%	1:8
**PNP2**	1:1			
**PNP3**	2:1			

PNP: polymeric nanoparticles.

**Table 2:** The encapsulation efficiency, drug loading capacity, particle size, zeta potential and polydispersity index of prepared DOXY-PNPs.

Formula Code	EE %	Drug Loading %	Particle Size (nm)	ZP (mv)	PDI
**PNP1**	42.15 ± 0.84	30.29 ± 0.15	203.6 ± 1.4	−21.8 ± 4.89	0.431 ± 0.02
**PNP2**	56.78 ± 0.52 ^£^	49.57 ± 0.11 ^£^	489.7 ± 6.7 ^£^	−23 ± 5.68	0.851 ± 0.05 ^£^
**PNP3**	84.65 ± 0.93 ^£,&^	66.13 ± 0.36 ^£,&^	615.3 ± 8.3 ^£,&^	−15.1 ± 4.84	1.00 ± 0.04 ^£,&^

EE%—encapsulation efficiency %; PDI—poly dispersity index. Data are mean ± SD and were analyzed using one-way ANOVA followed by Bonferroni’s post-hoc test at probability value < 0.05, ^£^ Compared to PNP1, ^&^ Compared to PNP2.

**Table 3:** Primers sequence and annealing temperature specific for each gene.

Target Gene	Primer Sequence: 5′-3′
*Caspase-3*	F: ATGTCAGCTCGCAATGG R: AAGAAATTATGGAATTG
*BAX*	F: CAGATCATGAAGACAGG R: GTGGATACAGACTCCCC
*GAPDH*	F: TAGGTATATGTTAAATTT R: GCTGACATTTAGGTAGAA
